# A Theoretical and Practical Treatise on Ligature of Arteries

**Published:** 1833-01-01

**Authors:** 


**Traité Théorique et Pratique de la Ligature des Arteres.** Par *J. P. Manec*, M.D. Premier Prosecteur à l'Amphithéatre Général des Hôpitaux Civils de Paris, &c. &c. Paris. Libraire Medicale de Crochard, Rue de l'Ecole de Medicine, No. 13. 1832.

It is somewhat singular, that, whilst the Medical Schools of England and the Continent have all afforded valuable works on anatomy, the subject of the present volume, at least as far as relates to its important illustrations, has been so much neglected. Whilst the greatest pains have been taken to represent the most minute, and comparatively, unimportant point in anatomy, and no expense has been spared to render these publications as correct as possible, as far as regards their immediate bearing on surgery, a blank still remained. It can be of little use to know the exact situation of parts individually, or indeed of their relation to one another, unless with this knowledge is connected a similar acquaintance with their relation to the skin which covers them; and, although in some works on operative surgery, rules are given as to the directions in which incisions are to be made, and the parts which are to serve as guides to these incisions, still something more was required; and that, as applicable to the ligature of arteries, is here supplied by M. Manec, a man who has acquired some reputation as an anatomist, from his beautiful plates of the sympathetic nerve.

The work commences by an introduction, containing much new and instructive matter on the structure of arteries, particularly on that of their fibrous coat, together with a general history of the ligature of arteries from the time of Desault until the present. The author next devotes considerable space to a consideration of the different effects and comparative danger of various ligatures—of the value of torsion of these vessels as a therapeutic agent, considered relatively to the effects of ligature—of the mode of placing a ligature on a vessel when affected by disease, whether of steatomatous or ossific deposits—and terminates by some general considerations on wounds of arteries, and the means adopted by nature to effect their occlusion. The operations of ligature are illustrated by very excellent plates, to which are appended descriptions of the various means which may be adopted for tying any vessel, although but one mode is represented in the drawing. Without any further preface, we will proceed to a more detailed examination of the work, in the form of analysis.

To take a view of the structure of arteries will be an excellent preparatory step to the consideration of the application of ligatures to them. The arterial system has been not incorrectly compared to a tree. Of its primary, secondary, ternary, and quaternary branches, none, with the exception of the mesenteric and vertebral interlace, but the anastomoses increase in number in proportion as we reach their ultimate subdivisions. The structure of arteries is not the same universally; or, if the same elements enter into their composition, they are singularly modified according to the capacity of the vessels. Arteries consist of three coats, a cellular, a fibrous (peculiar to arteries), and an internal, (similar to the internal tunics of veins). But besides these, the large arterial trunks are enclosed in a species of cellular tube, called their proper sheath. The external part of this sheath is blended with the surrounding cellular tissue; its internal is distinct from the cellular tunic of the arteries themselves. By extending an artery longitudinally, a separation is effected between it and the cellular sheath. But to vessels of the fourth or fifth order we find that this sheath no longer belongs, *e. g.* the radial, ulnar, tibial arteries, &c.

### EXTERNAL CELLULAR MEMBRANE.

In the large trunks this tunic lies in contact with the proper sheath, in the less, with the surrounding cellular membrane. Its internal surface adheres closely to the middle coat, the difference in their structure being the only line of separation; their union is effected by means of small cellular filaments passing from the outer coat to be inserted between the fibres of the middle coat. In examining some human aortæ, I have seen an intermediate tunic between the outer and middle, the nature of which I have not yet satisfactorily ascertained, although I should suspect, from its similarity to a structure in the same situation in the ox, that it is essentially glandular. The filaments of the cellular tunic are similarly interlaced to those of felt. Its vascular supply is of double origin—from surrounding vessels and from the artery itself. Extremely minute branches of the sympathetic nerve may be detected in it, by maceration for a few days in dilute nitric acid. We thus find that there is nothing peculiar in this membrane; not so in the next, or—

### MIDDLE FIBROUS TUNIC.

This is the thickest of the three membranes, but it is subject to variations in vessels of different sizes; it is very remarkable in the large trunks, and insensibly disappears in the small ones. Its colour also varies in different situations; it is yellow in the arch of the aorta; yellowish white in the iliacs and crural, and of a rosy tint in the smaller vessels. Its density is always in exact proportion to its thickness. This tissue (long considered as muscular) is formed of curved fibres, varying in length from one or two "millimétres,"* to two thirds of the circumference of the vessel. The most superficial of these fibres are the longest, their length gradually diminishing as they approach the internal tunic of the vessel, hence concentric layers are formed, the external of which are larger than the internal. This structure is easily detected by examining a transverse section of the aorta, by means of a magnifying glass. It may also be seen that the extremities of

---

* A millimétre is one thousandth part of a mètre, which is about 39 inches, English measure.

the curved fibres do not remain in the layer which is formed by them, but that they are inclined towards the internal tunic, to which they mostly adhere, by passing between the bundles of fibres placed beneath. The superficial fibres alone do not proceed in a direct manner, at least as far as can be well ascertained. They become confounded with the extremities of the deeper fibres, into which they are inserted. The ends of those fibres which form the internal layer of the middle coat, are immediately inserted into the internal tunic, but previously to coming in contact with this membrane they make a turn by which they change from a transverse to a longitudinal course. At the point where this turn is made the separate fibres cross one another so as to form a species of hook. It even appears that, after this crossing of the fibres upon each other, one of them takes one direction whilst another proceeds in a contrary course ; but I have never been able to follow a fibre throughout the whole of its course, and cannot speak with certainty as to this peculiar construction, although I am inclined to believe that such is the case. At the exterior of the middle coat the fibres are transverse and curved, at the interior they are longitudinal. This structure may easily be seen in the aorta of an ox, after maceration, if a magnifying glass be employed. The interlacement of fibres, just described, renders the internal portion of it very difficult to dissect, and gives to it that closeness of texture which it wants in other parts of its structure. From these observations it is evident, that the external surface of the middle tunic adheres to the cellular coat by the middle of the fibres of which it is formed, whilst its union with the internal coat is effected by means of the extremities of the same fibres. This disposition, whilst it gives great solidity to the internal face of the middle tunic, facilitates very much the movement of reaction which this membrane effects by its elasticity, after having been distended with blood. From some observations made on aneurisms in man, I was induced to make some experiments, which prove that the middle membrane alone can oppose any sanguineous infiltration, when the internal tunic has been by any means obliterated. The experiments consisted in destroying the internal tunic by means of a needle, when it was found that no blood penetrated the middle coat; but when the middle coat was torn by means of the needle, an infiltration took place, in two instances, as far as the surface of the vessel. The peculiar structure of the middle tunic is productive of a double result—1st, an augmentation of the elasticity of the fibres by the insertion of their extremities, the middle being left free; 2d, an insurmountable obstacle to sanguineous infiltration, even when the internal membrane is destroyed. The rare occurrence of aneurism in connexion with ossification of arteries, may be taken as a further proof of this last deduction; for in this case the blood must come into contact with the middle tissue, and yet aneurism is very rarely produced under such circumstances. The separate fibres of the middle tunic are united by a short cellular tissue which is readily ruptured by the longitudinal traction, but not so easily by torsion. In the very young, some vessels may be traced into this tunic, but it cannot be injected in later life. Nerves cannot be found in it.

### Internal Tunic.

This is connected with the middle coat by a tissue having no analogy with common cellular membrane. Its internal surface is similar to serous membranes, excepting that it has a somewhat velvety appearance. Its organization differs also from that of serous membranes, inasmuch as it is destitute of the extremely delicate interlaying filaments which characterize them. It is evidently formed of globular molecules, having all, one with another, the same connexions. Serous membranes are also composed of globular molecules, but arranged in such a manner as to form elementary fibres. The organization of serous membranes is of a higher character than that of the internal tunic of arteries; this latter is extremely small and readily torn, and cannot be regarded as a membrane, the end of which is to strengthen the parietes of the vessel; such is evidently not its purpose; it is merely to afford a more ready passage to the blood, and this is effected by an unctuous matter which moistens the internal surface. In experiments which I have tried on various animals to ascertain the function of the artery in secreting this substance, I have isolated a portion of an artery between two ligatures and allowed the blood to escape. After a certain number of hours, I have found a collection of this unctuous substance, varying in quantity and quality in proportion to the age and state of health of the animal, and very much influenced by the animal employed. In some other experiments, I denuded a portion of a vessel of part of its internal coat, thus leaving the middle coat naked in one or more places; from this, also, a secretion of unctuous matter took place, varying in quantity and quality according to the time which the experiment had lasted—thus, six or eight hours after the experiment, the whole of the unctuous matter was of a similar character; but in 36 or 48 hours this similarity no longer existed, and the matter presented a different aspect and different qualities, according as it was situated over the middle or internal membrane; over the former it was larger in quantity, adhered more firmly to the membrane, and was much more consistent than at any other part. Hence we may conclude, 1st, that the internal membrane alone does not afford unctuous matter; 2d, that the middle tunic contributes to its production; 3d, that when formed by the middle membrane it appears more plastic and coagulable. It is not improbable, from the plastic nature of this effusion from the middle tunic, that the internal coat may be re-produced by it, when by any cause it has been destroyed. Neither vessels or nerves can be detected in this internal coat, in its physiological state. Of the three tunics of arteries, the internal and middle are the most readily torn by moderate traction or pressure; the external alone resists the immediate action of constrictive force, and does not give way to longitudinal traction until some time after the others have been torn through.

After having devoted so much attention to the intimate structure of arteries, the author next proceeds to the more immediate subject of his work.

The phenomena presented by an artery to which a ligature has been applied, are dependent on, 1st, the size, form, and nature of the instrument employed; 2d, the size and condition of the vessel. The introduction of, and improvement in, the use of the ligature have gradually followed the plan employed by Desault to stop the current of the blood in an artery. The principles on which this effect is now produced were not at that time understood, and a variety of means were employed by celebrated surgeons, such as Bichat and Scarpa, without much success. No satisfactory improvement took place in this branch of surgery until 1805, when Dr. Jones published a valuable work on the means which nature adopts for the suspension of hæmorrhage. Various ligatures had been employed before the publication of this work, in which it was proved that the one in which the greatest confidence might be placed, was a small round ligature. Abernethy, Astley Cooper, and other eminent English surgeons adopted the practice, and with great success. It was not known in France until 1814, and was then so slightly spoken of by Roux, as to fall into disrepute. M. M. Beclard and Breschet repeated Jones's experiments, and with results so favourable that it was adopted and approved of by Dupuytren and Larrey; many, however, still continued to employ the broad, flat ligature, and do so even at the present day. It is to decide this question that M. Manec has paid a lengthened attention to, and tried numerous experiments on the subject, and in the hope that his endeavours will prove of service to mankind, and an assistance to the judgment of his professional brethren.

### General Principles.

In effecting the obliteration of an artery, the first indication is to suspend the passage of the blood; this may be effected in various manners—1st, by simply compressing the vessel and bringing its internal coat into close contact, in all its parts; 2d, by the same contact with more or less extensive rupture of the inner and middle coats; 3d, by a uniform compression of the artery, with a neat and regular section of its internal and middle coats.

M. Manec here enters into a description of the operation for ligature, as usually performed, and of the instruments which are necessary. The points particularly insisted on, are, the necessity of making the first incision sufficiently long (it is better that it should be too long than too short, because, in the latter case, the operation is more dangerous and tedious from the difficulty of reaching and of isolating the vessel; there is, also, much more cause to fear the effects of suppuration); the impropriety of opening the sheath more than is absolutely necessary to separate the artery from its accompanying vein and nerve; the necessity of avoiding all violent traction of the artery in surrounding it by its ligature, in which case its vessels of supply may be cut off, and death of the part take place. When the separation has been effected with violence, the artery at that part is only kept alive by continuity with itself; and if the patient be not young and strong, and the operation has been laborious, there is great probability that union will not take place between the artery and its sheath, but that a suppurative inflammation will ensue. Even if suppuration do not take place, still the violent separation of the vessel retards the succession of phenomena which should succeed one another before a perfect cure is effected. When an artery which is to be tied is situated close to a vein, the needle carrying the ligature should be introduced between the two vessels and carried outwards; the chance of wounding the vein is here very much less than it would be, were the needle passed in an opposite direction. If the vein be behind the artery, the latter should be gently elevated, and the needle carefully slid along its posterior surface. The same precautions are necessary in separating the artery from any nerves with which it may be in contact. A precautionary measure of great importance is, that when the knot is tied in the ligature, ths artery must not be elevated ; if it be situated deeply, after having drawn the first knot near the vessel, the two thumbs or fore-fingers should be placed between the threads, which are then to be drawn in opposite directions, by applying one nail against the other. When the first knot is made, the assistant should prevent its slipping with his finger, whilst the second is repeated in the same manner as the first, and this will be sufficient. The object, after the operation, should always be to obtain union by the first intention, one end of the ligature being cut off, and the other allowed to hang out of the wound. In order to ascertain whether any substance might be employed as a ligature, both ends of which might be cut off, and the wound closed over it, I have employed a variety of animal preparations, but in every experiment, inflammation and suppuration have followed, until the foreign body was discharged. This opinion is not shared by some surgeons of eminence, but such has been the result of all my endeavours, and the experience of M. Dupuytren has confirmed him in a similar opinion. Frequently two or three abscesses form in succession before the ligature is discharged.

Having thus described the operation, the author next proceeds to a consideration of the phenomena which shew themselves both in and around the artery, under the action of a variety of ligatures.

### Description of the Effects of a small round Ligature.

The internal and middle tunics of the artery are neatly and regularly divided; the cellular coat resists the action of the ligature, and is, consequently, brought into contact all around, being situated between the superior and inferior lips of the divided tunics. The cavities of the artery, above and below the ligature, are of a conical shape; the summits of these cones, formed by the lips of the divided membranes in contact with each other, oppose the infiltration of the blood into the cellular tunic. The pressure of the ligature is however completely confined to the cellular coat. The lips of the cut internal and middle membranes are kept in contact by the pressure of the external tunic, inclining them towards the centre of the vessel. This pressure, quite sufficient to close the artery, is not capable of destroying the vitality of the middle internal tunics. On the contrary, the cellular coat, on which alone the pressure is exercised, dies in that portion which is tied, by a suppurative inflammatory process. In about eight or ten hours after the application of the ligature, an exudation of concrete albumen or coagulable lymph takes place from the cut edges of the two membranes, which unites them to each other; this matter gradually increases in quantity and consistence; the column of blood forced against the part commences to deposit some filaments which form the foundation of a coagulum; the coagulum combines with the coagulable lymph, they unite together the borders of the division, but in so feeble a manner, that the least motion would suffice to separate them. But this is not the case twenty-four hours after the operation; this lymph has considerably increased in quantity, and has acquired a certain degree of organization; filaments are formed of sufficient strength to retain the lips in unison; such a condition of the parts as this seldom takes place before the fifteenth or twentieth hour. As the organization advances, the clot increases in size, and though at first deposited in little

"mamelons" becomes eventually a single coagulum. At this period the calibre of the artery is imperfectly filled by the clot, which contracts after its first formation, and by the passing to and fro of the blood in the interval between it and the artery, it suffers continual motion; gradually, however, a deposition of new matter in this interval increases the coagulum until the artery is completely stopped. The development of the coagulum is always first in length, and afterwards in breadth. This being the case, it sometimes happens, that when the ligature is applied too near a large collateral branch the coagulum does not acquire sufficient size to fill the vessel, except in a small extent, whilst at the other part it remains small; at other times the clot remains free and floating in its whole extent, but the force which the blood exercises in passing between it and the internal surface of the vessel is not sufficient to detach the unctuous matter, which under irritation is more abundantly secreted, and forms, as it were, a considerable layer. This unctuous matter serves to unite the clot to the vessel, as soon as, by their mutual increase, they come into contact with each other. The moment at which this union happens is various, but at whatever time it does take place, it is at first extremely feeble, soon, however, acquiring an evident consistence; at this period, organization and a change of appearance take place. The age of the individual, and the state of his constitution, are circumstances by which the rapidity or slowness of the organization are materially influenced. Youth and the adult age, or the sanguineous temperament, are favourable circumstances for rapidity of union, and vice versâ.

The growth of the coagulum is from its centre to its circumference, but its organization is in a retrograde direction, and the rapidity with which this takes place is in direct proportion to the active vitality of the vessel;—will be influenced by constitutional and local causes, such as an extensive separation of the artery and its sheath, by which it loses its chief source of nutrition. The first traces of organization begin to appear about six hours after the union of the clot and unctuous matter effused by the internal membrane; this organization is indicated by a filamentous appearance, soon afterwards becoming areolar; the whole tissue becomes lamellated, but before the central portions become thus organized, some red stripes appear in the parts nearest to the artery. These are, I suspect, absorbent vessels taking away the colouring matter of the blood; when no colouring matter remains, these striæ lose also their colour, and become much more solid and resisting. Although the commencement of organization in the clot is so soon after the operation, its completion is frequently not before six or eight weeks, or even more. But while these changes are going on within the artery, others of a more simple nature are taking place in the situation of the ligature. In three or four days after its application, inflammation of the cellular tissue sets in, which, if the ligature be small and round, will be of the adhesive kind, and will tend to unite the superior and inferior extremities of the artery, which are brought into apposition by the effects of the ligature; the exception to this union is only the situation of the knot. The extension of this inflammation is ordinarily two or three lines on each side of the ligature; suppuration ensues, the first effect of which is to destroy the albuminous tissue which united the ends of the artery, and eventually the portion of the cellular tissue which was embraced by the ligature dies, and is discharged with it. The inflammation affects both ends of the artery; the adhesions which united the base of the clots to the lips of the divided membranes are destroyed, the first measures adopted by nature to obliterate the vessel are thus done away with, and there remains but the clot and its connexion with the internal membrane, through the medium of the unctuous matter secreted, to save from consecutive hæmorrhage; hence the necessity of a large clot, in order to ensure a successful result to the operation, and hence the danger of proximity to a large collateral branch. This applies to operations on large vessels, or on those of middling size; when a smaller branch has been tied, a large clot is not of so much importance, as these branches appear to possess a greater degree of vitality, and, consequently, a more intimate union is effected. If a clot be not formed, hæmorrhage follows the suppurative process of the cellular coat. The same will take place if a violent inflammation extend to the coats of the artery, and thence to the coagulum.

### OF LARGE, FLAT LIGATURES.

The application of these ligatures produces a singular modification of the phenomena which follow the operation. The parietes of the vessel are brought together with violence, otherwise the internal and middle membranes would not be divided; and when this is effected, it is so by a severe laceration of them, parts of them being torn off, so as to remain within the cellular membrane compressed by the ligature. In such a case, the divided membranes do not turn in upon one another, and are, consequently, ill situated for effecting adhesion. The coagulable lymph is effused outwards, instead of in a situation to come in contact with the clot. The union of the two ends of the artery cannot take place, in consequence of the distance of their separation by the ligature. The plastic lymph being effused without the cavity of the internal membrane, this membrane loses one means of adhesion, and there remains only that afforded by the unctuous matter which it secretes, and which is formed more rapidly when the small, round ligature is employed. The union of the clot with the lips of the divided membrane fails from the absence of lymph; on account of the want of this "point d'appui," the clot is formed much later, and increases much more slowly than in the former instance, perhaps on account of its greater mobility. But the other processes, which ought to be slower, are accelerated; the inflammation is more rapid and violent, on account of the size of the ligature; the suppuration is extensive and long-continued, so that it is probable that the greater quantity of the adhesion between the clot and the internal surface of the artery will be destroyed by the inflammatory process. Suppose, also, that the same distance existed between the ligatures and a large collateral branch in two operations, one performed with the small, and the other with the broad ligature—the advantage is greatly in favour of the former. But if other circumstances favour, such as the advanced age of the patient—diseased tunics of the artery, its isolation from the sheath in a considerable extent; all of which tend to retard adhesion, hæmorrhage is certain. If there be no large collateral branch to interfere with the formation of a clot, it is not impossible that, after a partial destruction of the first, another *may* be formed, but this must depend entirely on the favourable situation of the ligature with regard to collateral branches, and other circumstances in the condition of the patient. Here we have another proof of the importance of applying a ligature as far as possible from collateral branches—an injunction which cannot be too strongly insisted on.

*The Operation performed with the Interposition of a Foreign Body between the Ligature and the Artery.* The object of this mode of treatment (first imagined and employed by Scarpa) is only to stop the current of blood in a vessel, in which, it was supposed, consisted the whole art of effecting a permanent occlusion. The varieties of "prope-artères" at present known, are all modifications of Scarpa's instrument. What has already been said of the danger of foreign bodies in ligatures of arteries, of the contusion arising from the forcible application of large ligatures, applies more strongly to the present than to the preceding form of ligature. By Scarpa's method, if any object beyond the mere contact of the internal coat is attempted, a more irregular and contused separation of the two tunics is effected than by the flat ligature. But Scarpa, in his latest essay on the subject, has so far confessed the evil of his former plan, as to give rules for the application of the broad ligature alone, without the use of a compress, as was his original plan.

The limited adoption of the small ligature on the Continent, and the preference still given by some surgeons to the coarse and dangerous employment of Scarpa's instrument, and the tribe of "prope-artères," have induced M. Manec to discuss the matter more fully than it would be to our advantage to do, considering the total disuse of the instruments in this country, and the preference almost universally given to the employment of the small, round ligature; we shall, therefore, terminate this part of the subject with the author's conclusions from the facts and experiments which he has enumerated, *i. e.* that the small, round ligatures are preferable to large ones, with whatever modifications the large ones may be employed; and that, notwithstanding the success which has attended the employment of other ligatures in the hands of some celebrated surgeons, it would appear that their success would have been far more extensive, had they substituted the small ligature for those to which they gave a preference.

### Torsion of Arteries.

M. M. Amussat, Velpeau, and Thierry proposed, about the same time, the torsion of arteries as a means of arresting hæmorrhage. This is not the place to discuss the question of priority of discovery; we will examine the merits of the operation, and the preference which it deserves over that of ligature. Each surgeon has his peculiar mode of operating: Amussat and Velpeau have only twisted the arteries in capital operations, or wounds of these vessels; Thierry proposes its employment in cases of aneurism. In these cases, M. Thierry passes a solid instrument beneath the artery, with which he afterwards twists it. If the artery be cut across, M. Thierry seizes it with a pair of pincers, the size of which is in proportion to that of the vessel, and with this he rotates it on itself, performing five or six rotations for small ones, and ten or twelve for those of larger size. Velpeau employs an ordinary pair of dissecting forceps; he seizes the end of the vessel, isolates it carefully from the surrounding parts, holds it with another pair of forceps somewhat above its extremity, in order to fix it firmly, whilst, with the former pair, he exercises rotation of the extremity.

M. Amussat employs two pair of forceps, each so constructed as to hold the vessel firmly; with the first, he seizes the artery and draws it out, separating it from all its connexions; he then applies the second pair, the bite of which is round and without points, just above the extremity of the first forceps, presses it firmly, so as to divide the internal and middle membranes, and, when he supposes the division to be complete, he pushes the second pair upwards, in order that the two divided tunics may turn in on one another. At this moment, M. Amussat retains the second pair firmly fixed, whilst, with the first pair, he rotates the cellular membrane of the vessel, which alone has resisted the action of the second forceps. By these various means, the current of the blood is stopped; in the method of Velpeau and Thierry, by an obstruction formed by the ruptured middle and internal coats; in that of M. Amussat, by the reflexion of the same membranes upon each other. Thus, then, the current of blood is stopped; but this is not the whole question, otherwise a ligature would answer the purpose better than any other mode of operating. The question of importance is—does torsion of arteries diminish the danger of consecutive hæmorrhage? M. Amussat considers that this question may be answered affirmatively: because, says he, there is no foreign body left in the wound, but little danger of inflammation and suppuration, and the external wound very readily cicatrizes. But M. Velpeau, who has made very numerous experiments on this mode of operating, considers the cases for its application as extremely limited, and that, even in those, it has no real advantage over the ligature. In animals, *e. g.* dogs and rabbits (he has not tried it in man), he has found the subsequent inflammation longer and more violent than in the cases of ligature. Such an occurrence is readily explicable, for torsion is a more tedious, painful, and dangerous operation than ligature. We will examine the question as it affects the operation on the extremities of vessels, as after amputation. When it is desired to apply a ligature, a very slight traction of the artery suffices; but for torsion of the same vessel, it would be necessary to draw it five or six lines beyond the flesh; the vessel, in this state, must then be isolated from the veins and nerves which accompany it, a precaution scarcely necessary in ligature. Again, as torsion destroys a larger portion of the vessel than ligature (in the case of vicinity to a large collateral branch), there is greater danger of a clot of insufficient size being formed, than when a small round ligature has been employed. But are the inconveniences of the operation compensated for by the advantages of its sequel? Far from it; inflammation and suppuration are as long, if not longer, than after ligature, and this may be explained in considering the length of the operation, the displacement of the connexions of the vessel in drawing it out, and the presence of a foreign body, *i. e.* the end of the twisted artery, the vitality of which is destroyed by the pressure of the forceps, and which cannot be separated excepting by the joint operation of inflammation and suppuration, both of which are as great as those produced by the knot of a ligature. The operation of torsion is far more applicable to small than to large vessels; in the former case it is less tedious, the vessel is destitute of a proper sheath; a fewer number of turns are required, on account of the smallness of the vessel, hence it is not always killed in the step of the process; but its use must be limited to cases of this sort, whilst at the same time, even here, it can receive no greater commendation than the small, round ligature.

### Ossified Arteries.

Besides these artificial means of stopping the current of the blood, there are some morbid processes which, in different degrees, produce a similar effect; such are, the production of cartilaginous and ossific deposits between the internal and middle membranes, and a squamous or steatomatous convex view of the middle tunic. These two conditions are very disadvantageous in the use of ligatures, even when they do but begin to appear; and when they are more advanced, success in the operation is almost impossible; the middle and internal tunics have lost their vitality by the morbid changes, and the cellular coat alone is left to support the current of the blood; and this tunic sooner or later gives way, unless the formation of a coagulum be very much favoured, by the distance of a collateral branch. It becomes a matter of importance to decide on the best means to be pursued in these cases. If a small, round ligature be employed, the osseous plates are broken down into irregular fragments; the uniform pressure of the cellular coat, so advantageous in a healthy condition of the parts, is here prevented, and there is substituted for it an irregular and uneven pressure; in this state, a very slight inflammation and suppuration suffices to destroy any adhesions, and the formation of a clot and its agglutination are consequently prevented. For these reasons, the small ligature should be, in all such cases, proscribed. The only applicable mode appears to be, equable, moderate, well-regulated pressure. In these cases, from the change in the organization of the middle and internal tunics, no assistance can be expected from them in performing their normal offices; the cellular tunic alone remains, and it is necessary to protect this, as long as possible, from the effects of inflammation and suppuration. From these circumstances, it would appear that the method advised by Bayer is the most applicable, that of keeping up a moderate pressure, insufficient to destroy the vitality of the cellular membrane. But when an ossified vessel is found in an amputated stump, such a means cannot be employed, as too great irritation would be thereby produced. In these cases, the method of Dupuytren and Roux, of introducing a piece of bougie into the vessel, and over that an ordinary ligature, is the best. An inflammation of considerable violence often supervenes, and the effect is so considerable a destruction of the clot, that if it be not some distance from a collateral vessel, hæmorrhage must be the inevitable effect. Of course, in these morbid states of arteries, torsion is totally inapplicable.

### Wounds of Arteries.

When an artery is completely divided, the cut ends retire into their cellular sheath, to the extent of from four or five lines to one inch. This contraction of the vessel, together with the feebleness induced by hæmorrhage, puts a stop to it, by closing almost entirely the mouth of the vessel. The effect of this is, the deposition of a coagulum within the vessel, which, in its organization follows the same laws as those which are formed after the application of ligatures. Thus, the spontaneous cure of hæmorrhage following an entire division of an artery, is as complete as if a ligature had been employed. If a longitudinal incision be made in the artery of an animal, the blood escapes at first with force; but this does not, as in the former case, extend to such a degree as to effect complete exhaustion of the animal. The first action which takes place in the wound, is the inversion of the middle membrane, caused by its elasticity; the cellular coat is left loose, and agitated by every successive fit of blood. The force of the current is thus much diminished, and an effusion takes place into the surrounding cellular membrane. This forms a multitude of little clots, which, together, acting as one coagulum, close the mouth of the vessel. If a semi-transverse incision be made into a vessel, the hæmorrhage which follows is with more difficulty arrested than in the last instance; the clot is formed in a different manner, and has a different shape. When the animal is very much weakened, and, from its exhaustion, the blood almost ceases to flow, a little clot forms around the lips of the wound, which, increasing from the circumference to the centre, finishes by its complete occlusion. This clot never acquires great thickness; it leaves the cavity of the artery quite free, as in the former instance, and a temporary cure is the effect. In the case of incomplete division of an artery, the cure is but apparent, for the artery is not obliterated, and the least disturbance of the part will produce a movement in the clot; in the first case, or that of complete division, a permanent cure is effected. With regard to the after-treatment of operations for ligature of arteries, enough is said in other works on operative surgery; I will only add, that it is requisite to take great care that too much blood is not abstracted, as the plethora which exists is quite necessary to force the blood in the capillaries superior to the ligature, into those which are inferior. A sanguineous evacuation can only be useful, where there are congestions towards any organ of importance.

We have thus terminated our analysis of M. Manec's work. The experiments from which the author has drawn his conclusions have been necessarily performed upon animals, and must, of course, be received with some modifications in their application to man; but when we consider the difference of phenomena which show themselves in animals and man, under a similar irritating cause, we shall find the arguments which have been adduced in favour of one plan of treatment much strengthened. The degree of inflammation which is produced by any irritating cause whatever, and the consequent constitutional sympathies, are always in proportion to the perfection of the organization. In proof of this, may be mentioned the slight effects produced in animals by operations and injuries, *e. g.* the gelding of horses, the spaying of sows, the after-effects of which are comparatively trifling, the fracture of bones, which, in dogs, is productive of little more inconvenience than that which arises from the difficulty of moving; and, as we descend farther in the scale of creation, we find abundant illustrations of the same general law, until we arrive at those animals whose propagation may be, as it were, effected by their division, *e. g.* worms. Thus, if, in an animal, the use of one ligature (the small, round one, for example,) produce a much less degree of inflammation than that of another (the broad, flat ligature), how much is the objection to the latter increased when the argument is applied to man, in which it is fair to conclude that the inflammation would acquire a proportionate increase. In the one case, the evil to be apprehended might be confined to that of the local inflammation; in the other, to the local affection might be added the danger of constitutional irritation; and thus, to state the case strongly, the selection of one or another ligature may be a choice between life and death.

